# EN2 Regulates Pancreatic Cancer Initiation, Progression, and Epithelial‐Mesenchymal Transition Through the Notch Signalling Pathway

**DOI:** 10.1111/jcmm.71158

**Published:** 2026-04-26

**Authors:** Wei Yu, Raj K. Varma, Yiming Ma, Varun Chandra Boinpelly, Areej Khatri, Emma Gombos, Rakesh K. Srivastava, Sharmila Shankar

**Affiliations:** ^1^ Kansas City VA Medical Center Kansas City Missouri USA; ^2^ Department of Cell and Molecular Biology Tulane University New Orleans Louisiana USA; ^3^ John W. Deming Department of Medicine Tulane University School of Medicine New Orleans Louisiana USA; ^4^ Southeast Louisiana Veterans Health Care System New Orleans Louisiana USA

**Keywords:** Bcl‐2, cMyc, cyclin D1, EN2, Nanog, notch, Oct4, pancreatic cancer, transformation

## Abstract

The incidence and mortality of pancreatic cancer are steadily increasing worldwide, and the disease is projected to become the second leading cause of cancer‐related deaths by 2030. Pancreatic tumorigenesis is driven by multiple genetic alterations, underscoring the need to elucidate the molecular mechanisms underlying pancreatic carcinogenesis. In this context, the present study is the first to identify a novel oncogenic role for Engrailed 2 (EN2) in the initiation and progression of pancreatic cancer and to characterise its underlying molecular pathogenesis. Immunohistochemical analysis of a pancreatic cancer tissue microarray revealed significantly elevated EN2 expression in tumour tissues compared with adjacent normal tissues. Consistent with these findings, EN2 was markedly upregulated in human pancreatic cancer cell lines, but absent in normal pancreatic epithelial cells, and functional studies demonstrated that EN2 expression is oncogenic in pancreatic cancer. TCGA data further corroborated the significantly higher EN2 expression in pancreatic cancer tissues and showed that elevated EN2 levels are associated with poor overall survival. To define the biological significance of EN2, we investigated its role in promoting pancreatic cancer initiation and progression. Gain‐ and loss‐of‐function studies revealed that EN2 regulates key target genes involved in pluripotency, cell survival, and cell‐cycle progression, drug resistance, and epithelial–mesenchymal transition. Moreover, lentiviral‐mediated shRNA knockdown of EN2 suppressed pancreatic cancer cell proliferation, invasion, and metastasis in vitro and significantly inhibited tumour growth in a xenograft mouse model, in part by inhibiting Notch signalling. Taken together, these findings identify EN2 as a critical driver of pancreatic cancer initiation, progression, and metastasis, representing the first report of its oncogenic function in this malignancy.

## 1 Introduction

Pancreatic ductal adenocarcinoma (PDAC), the predominant histological subtype, is currently the third leading cause of cancer‐related mortality in the United States. The incidence and mortality of pancreatic cancer continue to rise [1]. In 2025, an estimated 67,440 new cases were diagnosed in the United States, and approximately 51,980 individuals died from the disease [2]. The incidence and mortality of pancreatic cancer continue to rise, reflecting both an ageing population and the growing prevalence of risk factors such as obesity, diabetes, and chronic pancreatitis. Despite incremental therapeutic advances, PDAC remains one of the most lethal malignancies, largely because most patients present with advanced, unresectable disease and because effective early‐detection strategies are lacking [3, 4]. As a result, overall survival has improved only modestly, underscoring the urgent need for better biomarkers, earlier diagnosis, and more effective systemic therapies.

The engrailed family of genes encodes homeobox‐containing transcription factors, with Engrailed‐2 (EN2) belonging to the HOX gene family [5, 6, 7]. Hox genes, a highly conserved subgroup of the homeobox superfamily, play essential roles in embryonic development and regulate diverse biological processes, including apoptosis, receptor signalling, differentiation, motility, and angiogenesis [7, 8]. Dysregulated Hox gene expression has been implicated in abnormal development and numerous malignancies, suggesting that altered Hox activity can contribute to either oncogenesis or tumour suppression depending on cellular context. EN2, in particular, plays a critical role in embryonic development and stem cell self‐renewal [5, 6, 8]. Although aberrant EN2 expression has been reported in breast, bladder, and prostate cancers [5, 9, 10]. Its biological significance in these malignancies remains incompletely defined. Notably, EN2 has gained attention as a potential diagnostic biomarker, as elevated EN2 levels have been detected in the urine of patients with prostate and bladder cancer [9, 11, 12, 13, 14, 15]. TCGA analyses further reveal significantly increased EN2 expression in pancreatic cancer tissues compared with adjacent normal tissues. This differential expression suggests that EN2 may play a functional role in PDAC tumorigenesis and could serve as a potential biomarker for disease detection or progression. Elevated EN2 levels have been associated with oncogenic processes in several malignancies, and its upregulation in PDAC highlights the need for deeper investigation into its mechanistic contributions to pancreatic cancer biology.

Notch signalling is an evolutionarily conserved intercellular communication pathway that governs cell‐fate decisions during embryonic development and plays a critical role in maintaining tissue homeostasis in adult organisms [16, 17, 18]. Notch receptors are single‐pass transmembrane proteins that consist of three major regions: extracellular (NECD), transmembrane (TM), and intracellular (NICD) domains. Members of the Delta‐like (DLL1, DLL3, DLL4) and Jagged (JAG1, JAG2) ligand families bind to Notch receptors to initiate signalling [19, 20, 21]. Upon ligand engagement, the NECD is cleaved from the TM‐NICD fragment by TACE, after which the ligand–NECD complex undergoes endocytosis, recycling, and ubiquitination by Mib in the signal‐sending cell [20, 22, 23]. In the signal‐receiving cell, γ‐secretase cleaves the TM domain to release the NICD, which translocates to the nucleus and associates with the CSL transcription factor complex to activate canonical Notch target genes, including cMyc, Cyclin D1, and members of the HES family [18, 24]. CSL/RBPJ is a highly conserved DNA‐binding protein is responsible for cell fate decisions. In the absence of active Notch, CSL primarily functions as a transcriptional repressor, forming complexes with corepressors [25]. Upon NICD binding, CSL recruits a coactivator complex containing Mastermind‐like proteins (MAML1–3), thereby initiating a transcriptional program with broad phenotypic consequences [26]. Given that the Notch pathway is constitutively active in many cancers, it represents an important therapeutic target. However, the role of EN2 in regulating Notch signalling has never been demonstrated.

We investigated the role of EN2 in supporting the initiation and progression of pancreatic cancer. Our studies demonstrate that EN2 regulates key target genes involved in pluripotency (c‐Myc, Oct‐4, KLF4 and Nanog), cell survival and cell‐cycle progression (the Notch target gene Hes1 and cyclin D1), drug resistance (Bcl‐2), and epithelial–mesenchymal transition (E‐cadherin, N‐cadherin). Consistent with these molecular effects, lentiviral‐mediated shRNA knockdown of EN2 markedly suppressed pancreatic cancer cell proliferation, invasion, and metastasis in vitro and significantly inhibited tumour growth in a xenograft mouse model, in part by inhibiting Notch signalling. Notably, EN2 depletion reduced the expression of multiple Notch‐regulated genes, including Hes1, cMyc and cyclin D1. Taken together, these findings indicate that EN2 plays a critical role in the initiation, progression, and metastatic potential of pancreatic cancer, supporting its function as a novel oncogenic driver.

## 2 Materials and Methods

### 2.1 Cell Culture Conditions and Reagents

Human pancreatic cancer cell lines (PANC‐1 and AsPC‐1) and human normal pancreatic ductal epithelial cells (HPNE) were purchased from the American Type Culture Collection (ATCC, Manassas, VA). Pancreatic cancer cell lines were maintained in Roswell Park Memorial Institute (RPMI)‐1640 medium supplemented with 10% foetal bovine serum (FBS) and 1% antibiotic–antimycotic solution. Human pancreatic cancer stem cells (CSCs) were obtained from Celprogen and cultured in a well‐defined medium according to the manufacturer's instructions. Antibodies against Engrailed Homeobox 2 (EN2), hairy and enhancer of split‐1 (Hes1), and β‐actin were purchased from Cell Signalling Technology (Danvers, MA). Human pancreatic normal and cancerous tissues were acquired from the Cooperative Human Tissue Network (CHTN) and US Biomax (Derwood, MD). Enhanced chemiluminescence (ECL) Western blot detection reagents were obtained from Amersham Life Sciences (Arlington Heights, IL).

### 2.2 Lentiviral Particle Production and Transduction

The protocol for lentivirus production and transduction has been described by us previously [27, 28].

### 2.3 Spheroid Assay

Spheroid formation assays were performed as previously described [29].

### 2.4 Motility Assay

Scratch motility assays were performed as previously described [30].

### 2.5 Transwell Migration Assay

The Transwell migration assay was performed as described elsewhere [27].

### 2.6 Transwell Invasion Assay

Transwell invasion assays were performed as previously described [31].

### 2.7 Western Blot Analysis

Western blot analysis was performed as previously described [28]. Briefly, cell lysates were subjected to SDS–PAGE, and proteins were transferred onto nitrocellulose membranes (Amersham Biosciences, Piscataway, NJ, USA). After transfer, membranes were blocked with 5% skim milk or 5% BSA in Tris‐buffered saline (TBS) at 37°C for 2 h and then incubated overnight at 4°C with primary antibodies diluted 1:1000 in Tris‐Tween–buffered saline (TBS‐T). After three washes with TBS‐T, membranes were incubated with horseradish peroxidase–conjugated secondary antibodies (1:5000) for 1 h at room temperature, followed by three additional washes with TBS‐T. Protein–antibody complexes were visualised using enhanced chemiluminescence (ECL) substrate (Thermo Fisher Scientific, Rockford, IL).

### 2.8 Chromatin Immunoprecipitation (ChIP) Assay

Chromatin Immunoprecipitation assays were performed as we described elsewhere [32].

### 2.9 Quantitative Real‐Time PCR

Total RNA was isolated using the RNeasy Mini Kit (Qiagen, Valencia, CA). cDNA was synthesised from purified RNA using the High‐Capacity cDNA Reverse Transcription Kit (Applied Biosystems). Quantitative real‐time PCR was performed on an ABI 7300 Sequence Detection System using SYBR Green chemistry. Amplification conditions consisted of an initial denaturation at 95°C for 10 min, followed by 40 cycles of 95°C for 15 s and 60°C for 1 min. GAPDH was used as the endogenous control for normalisation. All assays were performed in triplicate, and relative mRNA expression levels were calculated using the ΔΔCt method. Fold changes in gene expression were calculated.

### 2.10 Immunohistochemical Staining

Tissue sections were incubated with the primary antibody (1:100) overnight at 4°C, followed by three washes in PBS. Sections were then incubated for 30 min at room temperature with a horseradish peroxidase (HRP)–conjugated goat anti‐mouse IgG secondary antibody [33]. Signal detection was performed using a goat anti‐mouse HRP‐polymer detection kit. Immunohistochemical staining was evaluated for staining intensity, with 0 = undetectable, 1 = weak, 2 = moderate, and 3 = strong.

### 2.11 Xenograft Experiment

Balb/c nude mice (6 weeks old) were purchased from the Jackson's laboratory (Bar Harbour, ME). All animal studies were performed under a protocol approved by the Institutional Animal Care and Use Committee (IACUC) at the Kansas City VA Medical Center (Kansas City, MO), in compliance with institutional and federal guidelines for the care and use of laboratory animals. AsPC‐1/Scrambled and AsPC‐1/EN2 shRNA cells (1 × 10^6^ cells mixed 1:1 with Matrigel) were injected into the dorsal left and right flanks of Balb/c nude mice, respectively. Experiment was performed for 45 days. At the end of the experiment, tumour volumes were measured and tumour tissues were stored for biochemical analyses.

### 2.12 Statistical Analysis

The mean and standard deviation (SD) were calculated for each experimental group. Differences between groups were assessed using one‐way ANOVA followed by Bonferroni's multiple‐comparison test, performed with PRISM statistical analysis software (GraphPad Software Inc., San Diego, CA). A *p* value < 0.05 was considered statistically significant.

## 3 Results

### 3.1 EN2 Is Differentially Expressed in Human Pancreatic Tissues, With Markedly Higher Levels Observed in Cancer Specimens Compared With Normal Pancreas

EN2 is a member of the HOX gene family and functions as a transcription factor that regulates embryonic development, pluripotency, and stem cell self‐renewal [34]. Recently, EN2 has been demonstrated to be implicated in the progression of some cancers, such as breast, prostate, and bladder [9, 14, 34, 35, 36, 37, 38]. However, no studies have examined the role of EN2 in pancreatic cancer. Therefore, we investigated EN2 expression and its association with pancreatic cancer progression.

**FIGURE 1:**
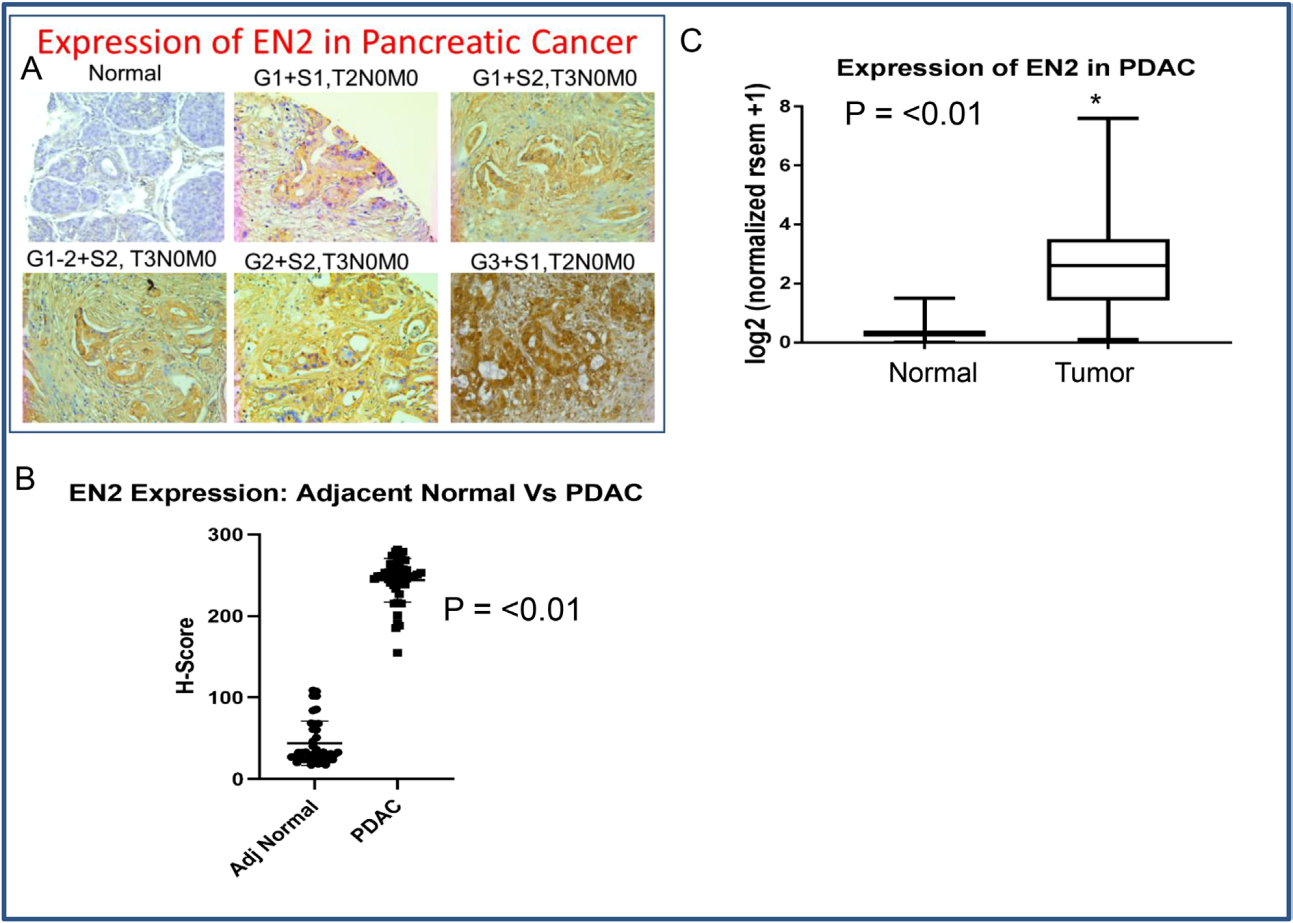
Expression of EN2 in human pancreatic cancer tissues. (A, B), Pancreatic Tissue Arrays containing normal and cancerous tissues were purchased from Biomax. EN2 expression was measured by IHC. Representative photographs of 60 pancreatic tissues from various stages of pancreatic cancer. Blue = nuclei, Brown/pink colour = EN2. * = significantly different from normal, p = < 0.01. (C) TCGA data on the expression of EN2 mRNA. * = significantly different from normal.

We first examined EN2 expression in normal and cancerous human pancreatic tissues. As shown in Figure 1A,B, EN2 is highly expressed in pancreatic cancer tissues, whereas its expression is either very low or undetectable in normal pancreatic ducts and adjacent non‐malignant tissues. Consistent with these observations, TCGA data also showed significantly higher EN2 mRNA levels in pancreatic cancer than in normal tissues (Figure 1C). Together, these findings indicate that EN2 is selectively upregulated in pancreatic cancer and suggest that it may serve as a useful diagnostic biomarker for the disease.

### 3.2 EN2 Is Highly Expressed in Pancreatic Cancer Stem Cells (CSCs) and in Established Pancreatic Cancer Cell Lines, Supporting Its Association With Stemlike Properties and Tumour Aggressiveness

**FIGURE 2:**
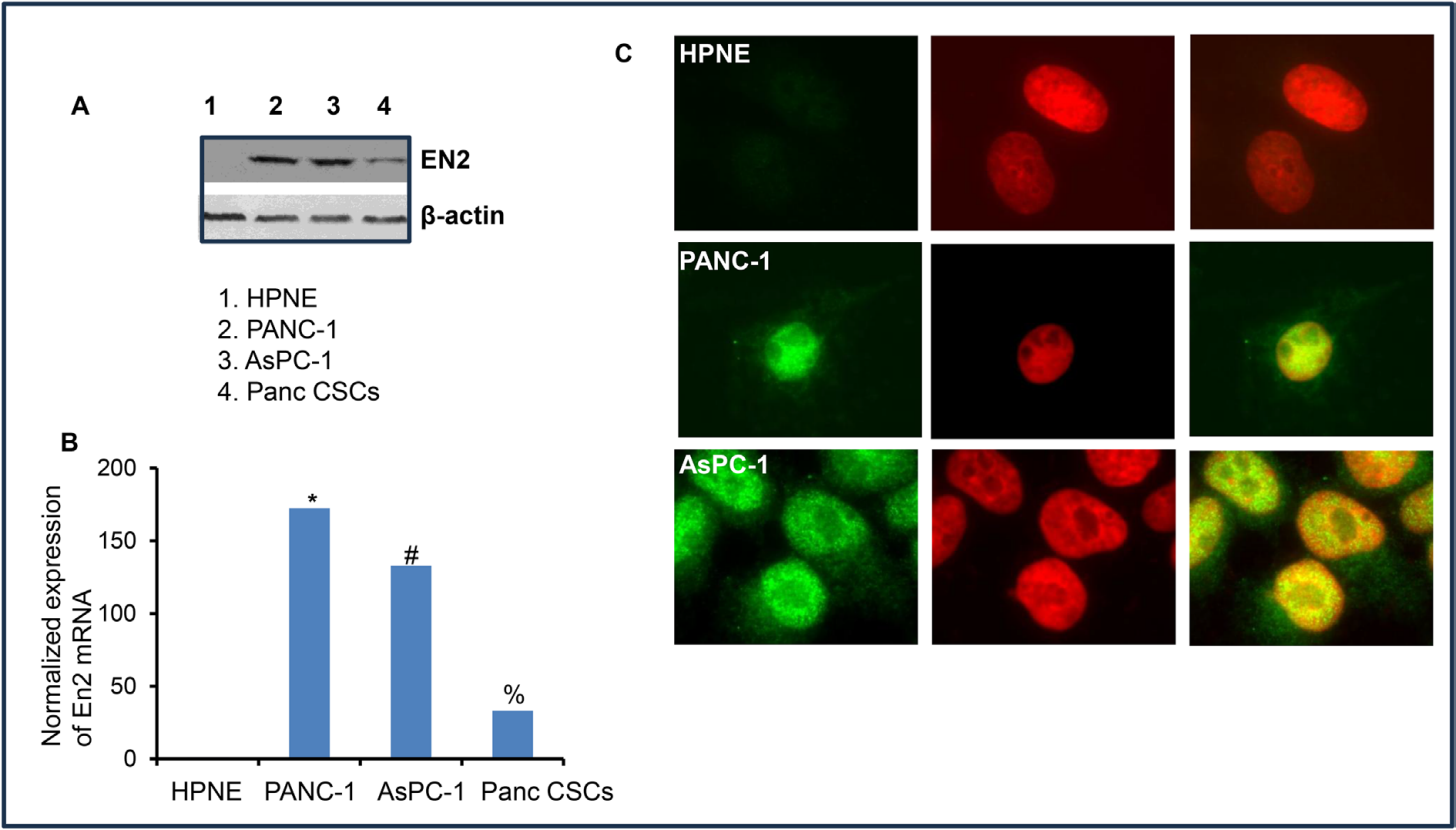
The expression of EN2 in HPNE, pancreatic cancer cell lines, and pancreatic CSCs. (A), Protein expression of EN2 in HPNE, pancreatic cancer cell lines, and pancreatic CSCs. Crude proteins were isolated, and EN2 expression was measured by Western blot analysis. β‐Actin was used as a loading control. (B), Expression of EN2 mRNA in HPNE, pancreatic cancer cell lines, and pancreatic CSCs. RNA was isolated, and EN2 expression was measured by q‐RT‐PCR. GAPDH was used as an internal control. Data represent mean (*n* = 4) ± SD. *, # and % = significantly different from HPNE (*p* < 0.05). (C), Expression of EN2. Immunocytochemistry was performed to examine EN2 expression in HPNE, PANC‐1, and AsPC‐1 cells.

We next determined the EN2 expression profile in pancreatic cancer cell lines and pancreatic cancer stem cells, and compared it with that of normal pancreatic epithelial cells. EN2 expression in PANC‐1, AsPC‐1, and pancreatic CSCs was measured by Western blot analysis, q‐RT‐PCR, and immunocytochemistry (ICC), using HPNE cells as a normal control. As shown in Figure 2A, EN2 is highly expressed in both pancreatic cancer cell lines and pancreatic CSCs, whereas HPNE cells exhibit minimal to no EN2 expression. These findings indicate that EN2 upregulation is associated with pancreatic tumour cells and is further enhanced in the CSC population, suggesting a potential role for EN2 in pancreatic cancer progression and stemness.

We next performed qRT‐PCR and immunocytochemistry to examine EN2 expression in human pancreatic cancer cell lines and CSCs and compared it to that of human pancreatic ductal epithelial (HPNE) cells. EN2 mRNA is highly expressed in human pancreatic cancer cell lines PANC‐1 and AsPC‐1 and CSCs and is not expressed in human normal pancreatic ductal epithelial (HPNE) cells (Figure 2B). Similarly, immunohistochemical data showed that EN2 is expressed in pancreatic cancer cell lines and CSCs but not in HPNE cells (Figure 2C). Our results demonstrate that mRNA and protein levels of EN2 were significantly higher in pancreatic cancer cell lines and pancreatic CSCs than those in HPNE cells. These data thus suggest that EN2 expression is tightly regulated in pancreatic cancer.

### 3.3 Overexpression of EN2 in HPNE Cells Promotes Cellular Transformation and Enhances Stemness, in Part by Upregulating Stem Cell–Associated Markers and Pluripotencymaintaining Factors

**FIGURE 3:**
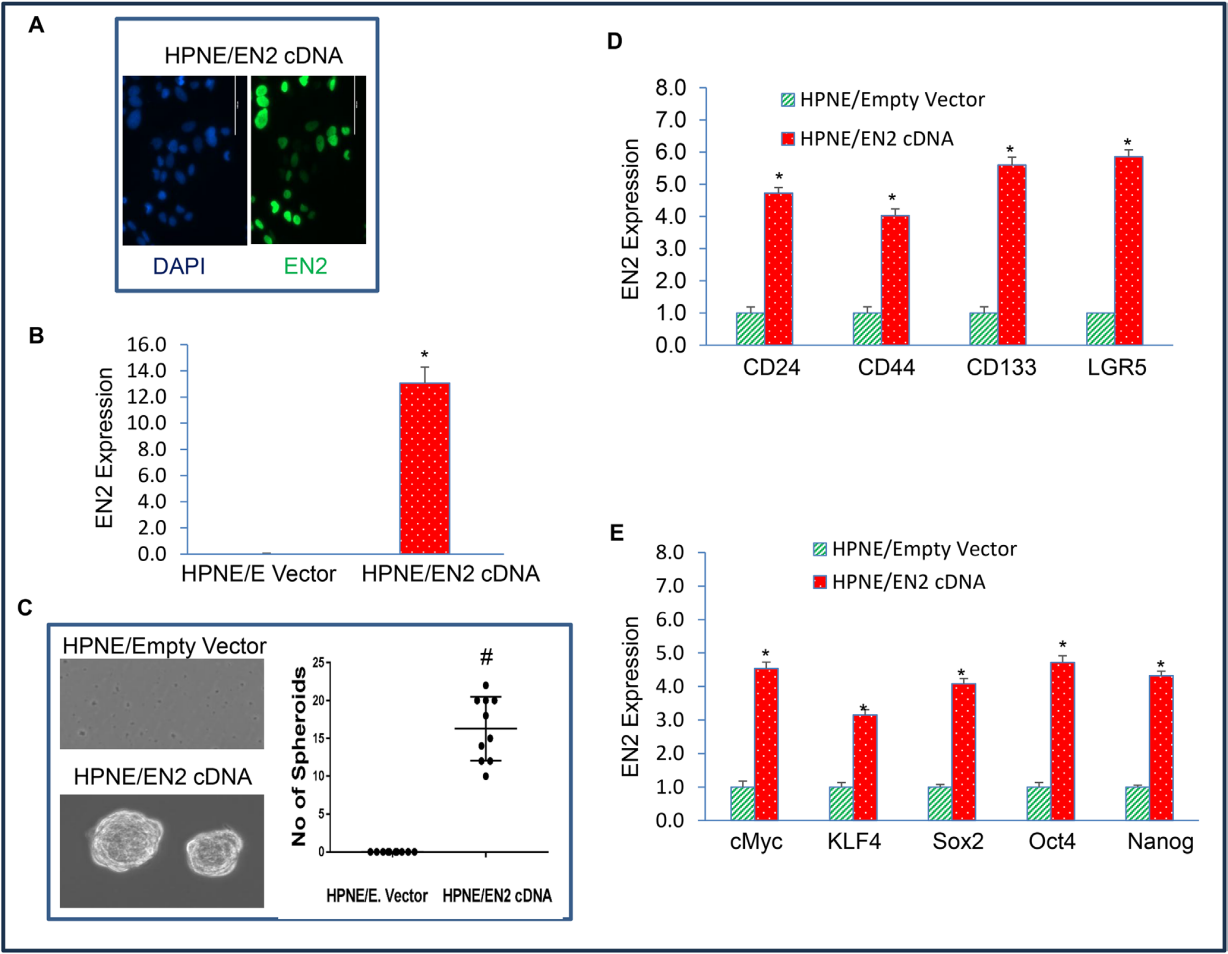
Overexpression of EN2 in HPNE cells induces cellular transformation and stemness. (A and B) HPNE cells were stably transduced with lentiviral particles expressing either empty vector or EN2 cDNA. EN2 expression was measured by immunocytochemistry and qRT‐PCR. Blue colour = nuclei; Green colour = EN2. Data represent mean (*n* = 4) ± SD. * = significantly different from HPNE/Empty Vector group (*p* < 0.05). (C), Spheroid formation in suspension. Spheroid formation of HPNE/Empty Vector and HPNE/EN2 cDNA cells was measured. Spheroids in suspensions were photographed (left) and counted (right). Data represent mean (*n* = 4) ± SD. # = significantly different between groups (*p* < 0.05). (D), Expression of stem cell markers. RNA was isolated, and expression of stem cell markers (CD24, CD44, CD133 and LGR5) was measured by qRT‐PCR. GAPDH was used as an internal control. Data represent mean (*n* = 4) ± SD. * = significantly different from HPNE/Empty Vector group (*p* < 0.05). Gene expression of Empty Vector was normalised to 1. (E), Expression of pluripotency‐maintaining factors. RNA was isolated, and the expression of pluripotency‐maintaining factors (Oct4, Sox2, cMyc and KLF4) was measured by qRT‐PCR analysis. GAPDH was used as an internal control. Data represent mean (*n* = 4) ± SD. * = significantly different from HPNE/Empty Vector group (*p* < 0.05). Gene expression of Empty Vector was normalised to 1.

The process of malignant transformation is the first step in oncogenesis and is characterised by high or indefinite saturation density, loss of contact inhibition, disorganised growth, reduced tight junctions, and colony formation [39, 40, 41]. To determine whether EN2 promotes cellular transformation and stemness, we overexpressed EN2 in HPNE wild‐type cells. Lentiviral‐mediated delivery of EN2 generated HPNE/EN2 cells that exhibited robust EN2 expression, as shown by immunocytochemistry (Figure 3A). In contrast, HPNE cells transduced with the empty vector (HPNE/Empty) showed no detectable EN2 signal (data not shown). EN2 overexpression was further validated at the transcript level by qRT‐PCR, which confirmed a marked increase in EN2 mRNA in HPNE/EN2 cells relative to controls (Figure 3B).

Normal epithelial cells do not form spheroids in suspension, whereas malignantly transformed epithelial cells readily acquire this ability [27]. HPNE/EN2 cells exhibited clear evidence of cellular transformation, as demonstrated by their capacity to form spheroids in suspension (Figure 3C). In contrast, the HPNE/empty vector group failed to form spheroids under the same conditions. These findings indicate that EN2 expression is sufficient to confer anchorage‐independent growth properties on HPNE cells, suggesting that EN2 can drive the malignant transformation of otherwise non‐tumorigenic pancreatic epithelial cells.

Malignant transformation is frequently accompanied by the emergence of cancer stem cell–like populations, defined by upregulation of stem cell markers and pluripotency‐maintaining transcription factors [27, 30, 42, 43]. Because EN2 overexpression in HPNE cells induced malignant transformation, we next examined whether EN2 also promotes a stem‐like phenotype. As shown in Figure 3D, EN2 overexpression markedly increased the expression of stem cell markers CD24, CD44, CD133 and LGR5, indicating the acquisition of stem cell‐associated characteristics. In addition, EN2 overexpression elevated the expression of key pluripotency‐maintaining factors, including c‐Myc, KLF4, Sox2, Oct4 and Nanog (Figure 3E). Together, these findings demonstrate that EN2 overexpression in HPNE cells not only drives malignant transformation but also induces a stem cell–like transcriptional program.

### 3.4 Overexpression of EN2 in HPNE Cells Enhances Cell Migration and Induces Epithelial‐Tomesenchymal Transition (EMT)–like Characteristics, Supporting a Role for EN2 in Promoting a More Motile and Mesenchymal Phenotype

**FIGURE 4:**
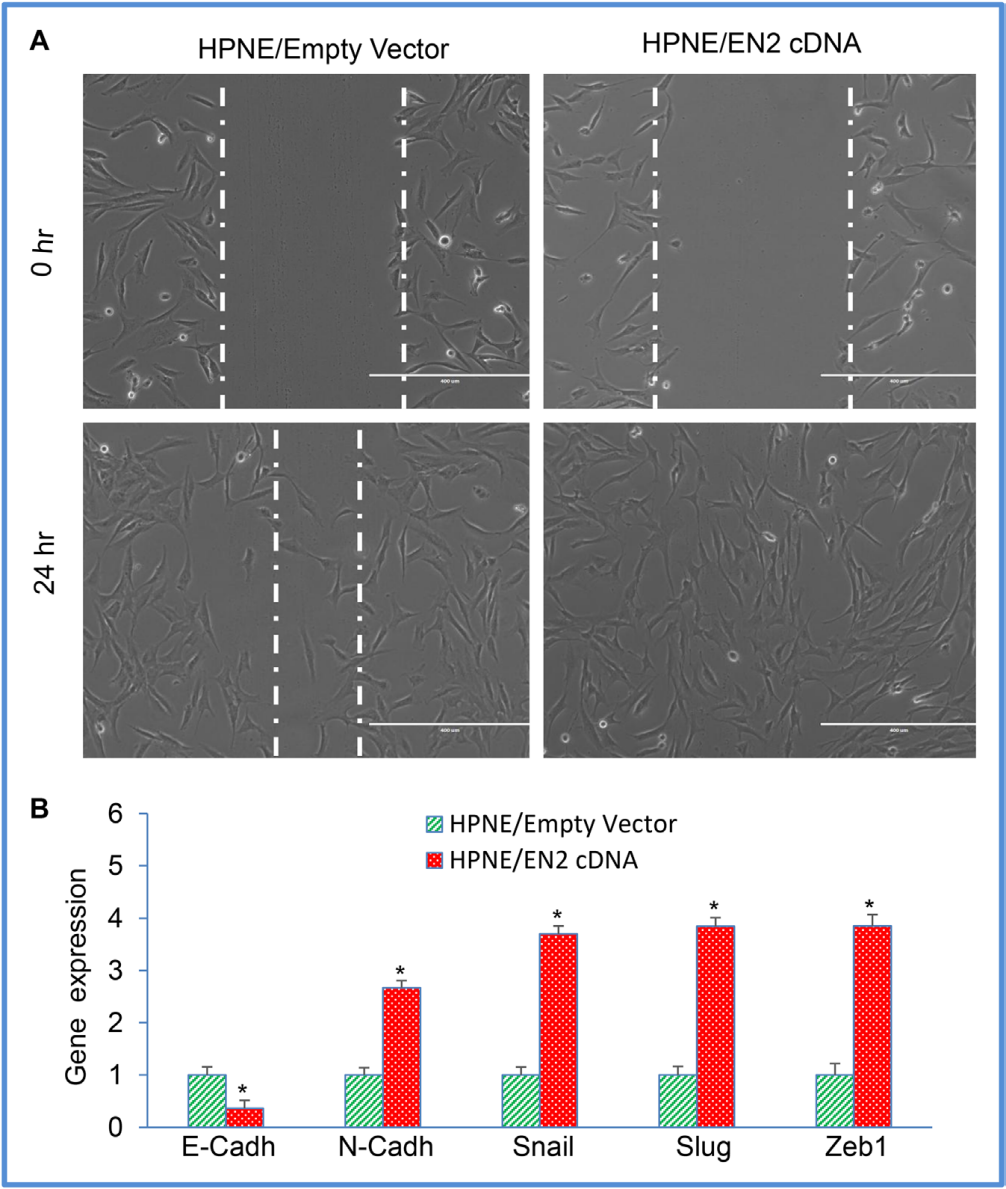
Overexpression of EN2 in HPNE cells enhances cell motility and modulates expression of EMT‐related genes. (A) Cell Motility. HPNE cells were stably transduced with lentiviral particles expressing either empty vector or EN2 cDNA. (B) Expression of EMT‐related genes. RNA was isolated, and the EMT‐related genes (E‐cadherin, N‐cadherin, Snail, Slug, and Zeb1) were measured by qRT‐PCR analysis. GAPDH was used as an internal control. Data represent mean (*n* = 4) ± SD. * = significantly different from HPNE/Empty Vector group (*p* < 0.05). Gene expression of Empty Vector was normalised to 1.

The cellular transformation of EN2‐overexpressing HPNE cells was further supported by their acquisition of migratory behaviour. As shown in Figure 4A, HPNE/EN2 cells exhibited significantly greater motility than HPNE/Empty vector controls, indicating that EN2 enhances cell movement—a hallmark associated with transformed phenotypes. Because EN2 overexpression increased motility, we next examined whether EN2 influences the expression of epithelial–mesenchymal transition (EMT)–related genes. EN2 overexpression reduced E‐cadherin levels while increasing N‐cadherin expression, along with the EMT‐associated transcription factors Snail, Slug, and Zeb1 (Figure 4B). These findings suggest that EN2 overexpression promotes EMT‐like characteristics in HPNE cells.

### 3.5 Knockdown of EN2 in Pancreatic Cancer Cell Lines Suppresses Cell Proliferation, Indicating That EN2 Contributes to the Growthpromoting Properties of These Cells

**FIGURE 5:**
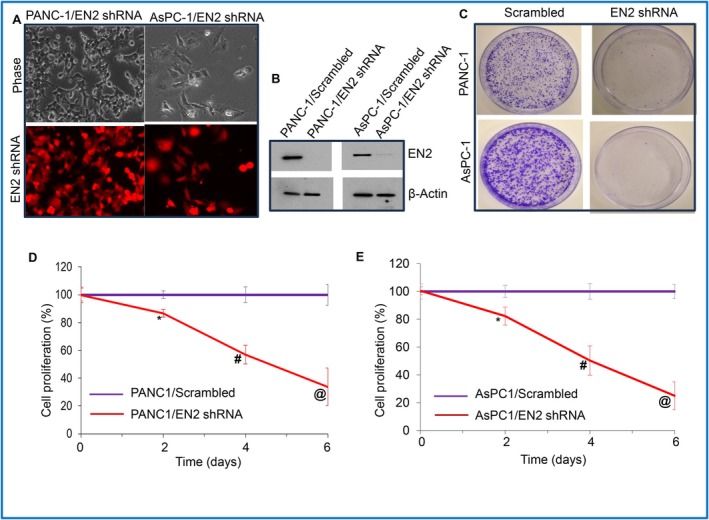
EN2 shRNA inhibits cell proliferation in PANC‐1 and AsPC‐1. PANC‐1 and AsPC‐1 were transduced with lentiviral particles expressing either Scrambled or EN2 shRNA (targeting four different sites). (A) Phase and fluorescence images of EN2 shRNA‐infected PANC‐1 and AsPC‐1 cells (verification of infection). (B) Western blot analysis was performed to assess EN2 expression. β‐Actin was used as a loading control. (C) Colony formation. PANC‐1 and AsPC‐1 cells infected with Scrambled and EN2 shRNA were seeded in petri dishes. After 21 days, images of the colonies were photographed. (D and E) Cell Proliferation. Cell proliferation was measured over a 6‐day period. Data represent mean (*n* = 4) ± SD. *, #, @ = significantly different between groups (*p* < 0.05).

Since EN2 overexpression induces cellular transformation in HPNE cells, we next tested the hypothesis that EN2 knockdown in pancreatic cancer cells would suppress cell proliferation and EMT. To assess the biological significance of EN2 in pancreatic cancer, we used a lentiviral vector encoding EN2‐targeting shRNA to reduce EN2 expression in PANC‐1 and AsPC‐1 cells. Successful infection was confirmed by red fluorescence in EN2 shRNA–transduced cells (Figure 5A). EN2 knockdown was validated at both the mRNA and protein levels using qRT‐PCR and Western blotting, respectively. As shown in Figure 5B, lentiviral delivery of EN2 shRNA markedly reduced EN2 expression in both PANC‐1 and AsPC‐1 cells.

We next evaluated the effects of EN2 knockdown on colony formation and cell proliferation in PANC‐1 and AsPC‐1 pancreatic cancer cells. EN2 silencing markedly reduced colony‐forming ability in both cell lines compared with scrambled shRNA controls (Figure 5C). Consistent with this finding, EN2 knockdown also resulted in significantly decreased cellular proliferation relative to scrambled controls (Figure 5D,E). Taken together, these results demonstrate that EN2 depletion suppresses the proliferative capacity of pancreatic cancer cells.

### 3.6 Knockdown of EN2 in Pancreatic Cancer Cells Inhibits Epithelial–Mesenchymal Transition (EMT), Indicating That EN2 Is Required to Sustain the Mesenchymal, Migratory Phenotype Associated With Tumour Progression

**FIGURE 6:**
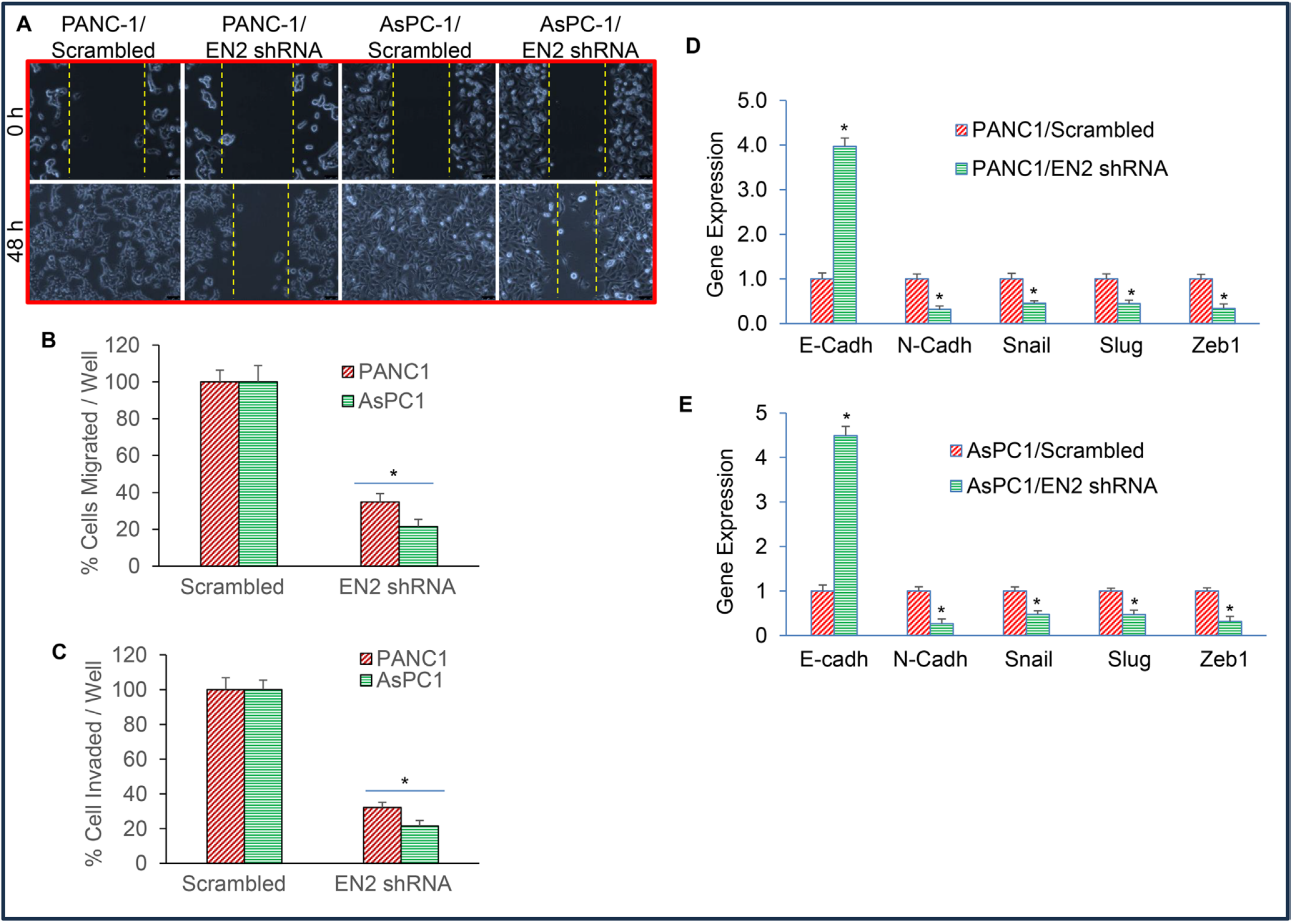
EN2 knockdown reduces motility, migration, invasion, and EMT marker expression in pancreatic cancer cells. (A) Cell Motility Assay. Pancreatic cancer cells expressing scrambled or EN2 shRNA were cultured in petri dishes. After 18 h, a linear scratch was generated using a fine pipette tip, and phase‐contrast images were captured at 0 and 48 h to assess wound closure. (B) Cell Migration Assay. Cells expressing scrambled or EN2 shRNA were seeded in six‐well plates, and migration was quantified as described in the Materials and Methods. Data represent mean (*n* = 4) ± SD. *p < 0.05 compared with the scrambled control. (C) Cell Invasion Assay. Cells expressing scrambled or EN2 shRNA were seeded in six‐well plates, and invasion was measured as described in the Materials and Methods. Data represent mean (*n* = 4) ± SD. *p < 0.05 compared with the scrambled control. (D, E) Total RNA was isolated, and the expression of E‐cadherin, N‐cadherin, Snail, Slug, and Zeb1 was quantified by qRT‐PCR. GAPDH served as the internal control. Data represent mean (*n* = 4) ± SD. *p < 0.05 between groups.

Epithelial–mesenchymal transition (EMT) is a process in which epithelial cells lose cell–cell adhesion and acquire enhanced migratory and invasive capabilities [44, 45]. Because EMT plays a central role in pancreatic cancer metastasis, we examined whether EN2 influences motility, migration, and invasion in pancreatic cancer cells. EN2 knockdown significantly reduced cell motility in both PANC‐1 and AsPC‐1 cells compared with their respective scrambled controls (Figure 6A). Consistent with this, EN2 silencing also inhibited cell migration and invasion in both cell lines (Figure 6B,C). These findings indicate that suppression of EN2 expression attenuates the motility and invasiveness of pancreatic cancer cells.

Since EN2 knockdown inhibited EMT‐associated behaviours, we next investigated the molecular mechanisms underlying this effect. E‐cadherin is a key epithelial adhesion molecule that maintains cell–cell contacts, and its loss is a critical initiating event in EMT. EN2 silencing increased E‐cadherin expression while reducing N‐cadherin levels in both PANC‐1 and AsPC‐1 cells (Figure 6D,E). Because cadherin switching is often driven by EMT‐associated transcription factors, we assessed whether EN2 knockdown affects the expression of Snail, Slug, and Zeb1. Transduction with EN2 shRNA markedly decreased the expression of all three transcription factors. Together, these findings indicate that EN2 inhibition modulates EMT by regulating both cadherin expression and key EMT‐related transcriptional regulators, thereby suppressing the invasive and metastatic potential of pancreatic cancer cells.

### 3.7 EN2 shRNA Inhibits the Expression of Genes Associated With Cell Proliferation, Cellcycle Progression, and Stemness, and Also Suppresses Components of the Notch Signalling Pathway

**FIGURE 7:**
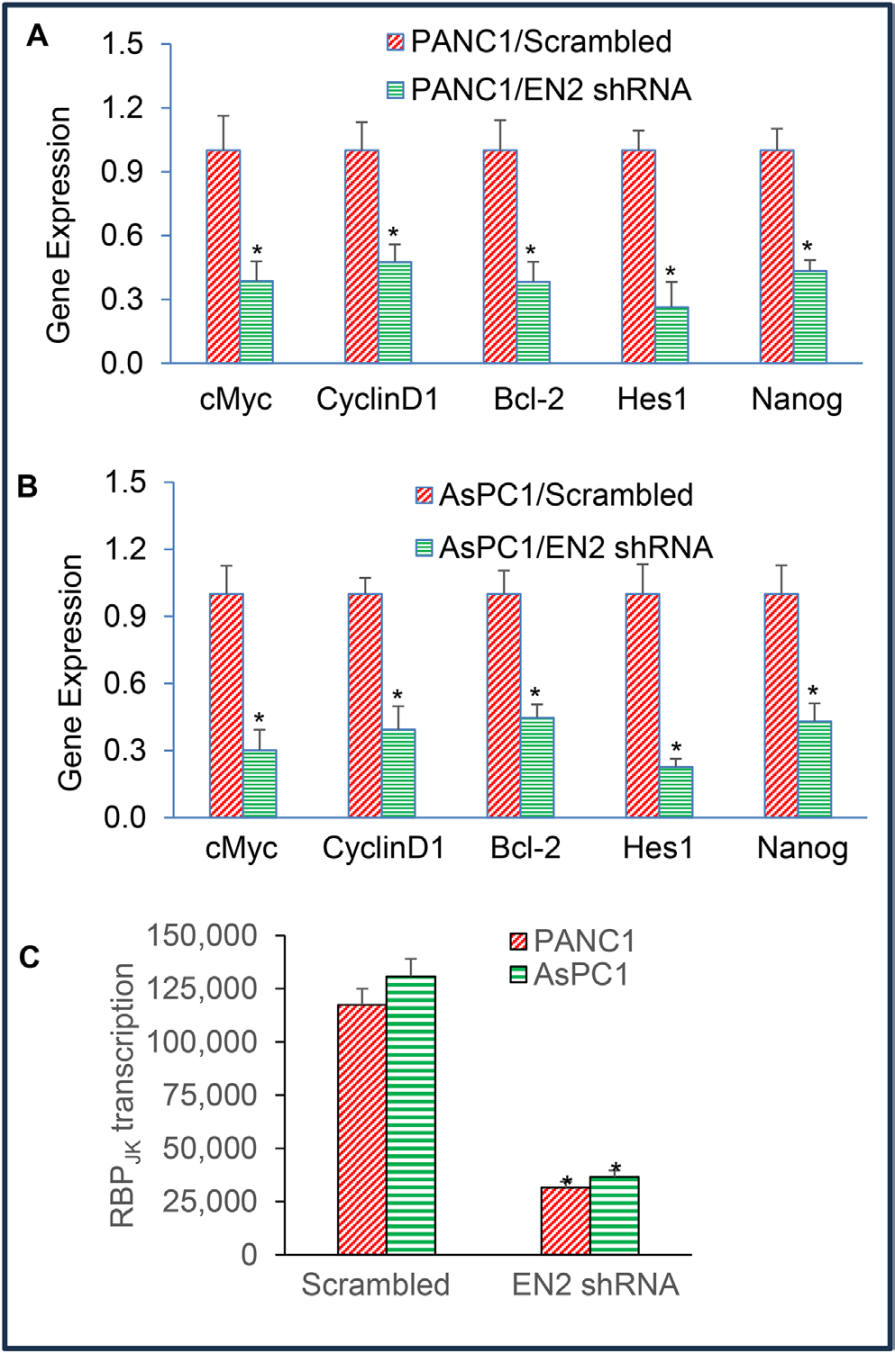
EN2 shRNA inhibits Notch‐target genes and Nanog expression and RBP_JK_ transcription in pancreatic cancer cells. (A, B), Expression of Notch target genes. RNA was isolated, and the expression of cMyc, Cyclin D1, Bcl‐2, Hes 1 and Nanog was measured in cells by q‐RT‐PCR. GAPDH was used as an internal control. Data represent mean (*n* = 4) ± SD. * = significantly different between groups (*p* < 0.05). (C), RBP_JK_ transcription. PANC‐1 and AsPC‐1 cells were transduced with RBP_JK_‐responsive GFP/firefly luciferase viral particles (pGreen Fire1‐ RBP_JK_ with EF1, System Biosciences) along with EN2/scrambled or EN2 shRNA viral particles. RBP_JK_ reporter activity was measured as we described [46]. Data represent mean (*n* = 4) ± SD. * = significantly different from scrambled control group (*p* < 0.05).

EN2 is a transcription factor known to regulate genes involved in cell proliferation, cell‐cycle progression, and stemness. Because c‐Myc, Nanog, and components of the Notch pathway are key regulators of stemness, we next examined whether EN2 knockdown affects their expression. As shown in Figure 7A,B, EN2 shRNA markedly reduced the expression of c‐Myc, cyclin D1, Bcl‐2, Hes1 and Nanog in both PANC‐1 and AsPC‐1 cells compared with scrambled controls. These findings indicate that EN2 regulates pathways governing proliferation, survival, cell‐cycle progression, and stemness in pancreatic cancer cells.

Since EN2 shRNA inhibited the Notch target gene Hes1, we next assessed whether EN2 regulates Notch signalling at the transcriptional level by measuring RBP‐Jκ transcriptional activity. EN2 knockdown significantly reduced RBP‐Jκ activity in both PANC‐1 and AsPC‐1 cells, indicating impaired Notch pathway signalling (Figure 7C). Together, these results suggest that EN2 promotes cell proliferation, survival, cell‐cycle progression, and stemness at least in part by activating the Notch pathway, further supporting its role as a central regulator of oncogenic signalling in pancreatic cancer.

### 3.8 EN2 Directly Binds Nanog, Bcl‐2 and Cyclin D1 in PANC‐1 Cells, Indicating That EN2 May Regulate Stemness, Survival, and Cellcycle Progression Through Direct Protein–Protein Interactions

**FIGURE 8:**
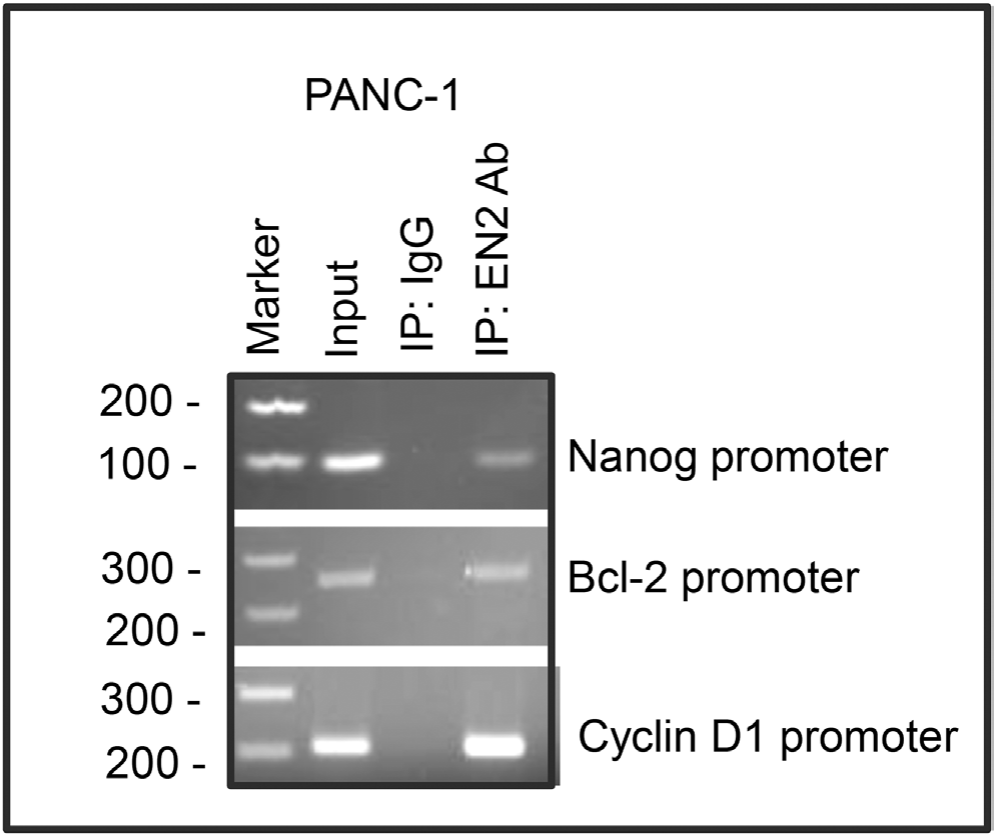
Binding of EN2 to the promoters of Nanog, Bcl2 and Cyclin D1. Chromatin immunoprecipitation (ChIP) was performed using nuclear extracts prepared from PANC‐1 cells to assess EN2 binding to the promoter regions of Nanog, Bcl‐2 and Cyclin D1. PCR products from the ChIP assay were analysed by gel electrophoresis. Lane 1: DNA marker; Lane 2: Input chromatin; Lane 3: IgG immunoprecipitation control; Lane 4: EN2 immunoprecipitation using anti‐EN2 antibody.

A chromatin immunoprecipitation (ChIP) assay uses an antibody to pull down specific DNA‐binding proteins, such as transcription factors, together with the genomic regions they occupy, enabling the identification of protein–DNA interactions in living cells. EN2 is a transcription factor known to regulate stemness, cell proliferation, and differentiation, and elevated Nanog expression in cancer cells is closely associated with tumour initiation, invasiveness, and therapeutic resistance. To determine whether EN2 directly regulates Nanog, we performed a ChIP assay. As shown in Figure 8, EN2 directly binds to the Nanog promoter, demonstrating a physical interaction between EN2 and this key stemness regulator. EN2 also binds to the promoters of Bcl‐2 and cyclin D1, indicating that its regulatory influence extends to genes involved in cell survival and cell‐cycle progression. Together, these findings suggest that EN2 orchestrates multiple cellular functions through direct transcriptional regulation of Nanog and other oncogenic targets, reinforcing its role as a central driver of pancreatic cancer biology.

### 3.9 EN2 Knockdown Suppresses Pancreatic Cancer Tumour Growth In Vivo, Demonstrating That EN2 Is Required for Efficient Tumour Progression

**FIGURE 9:**
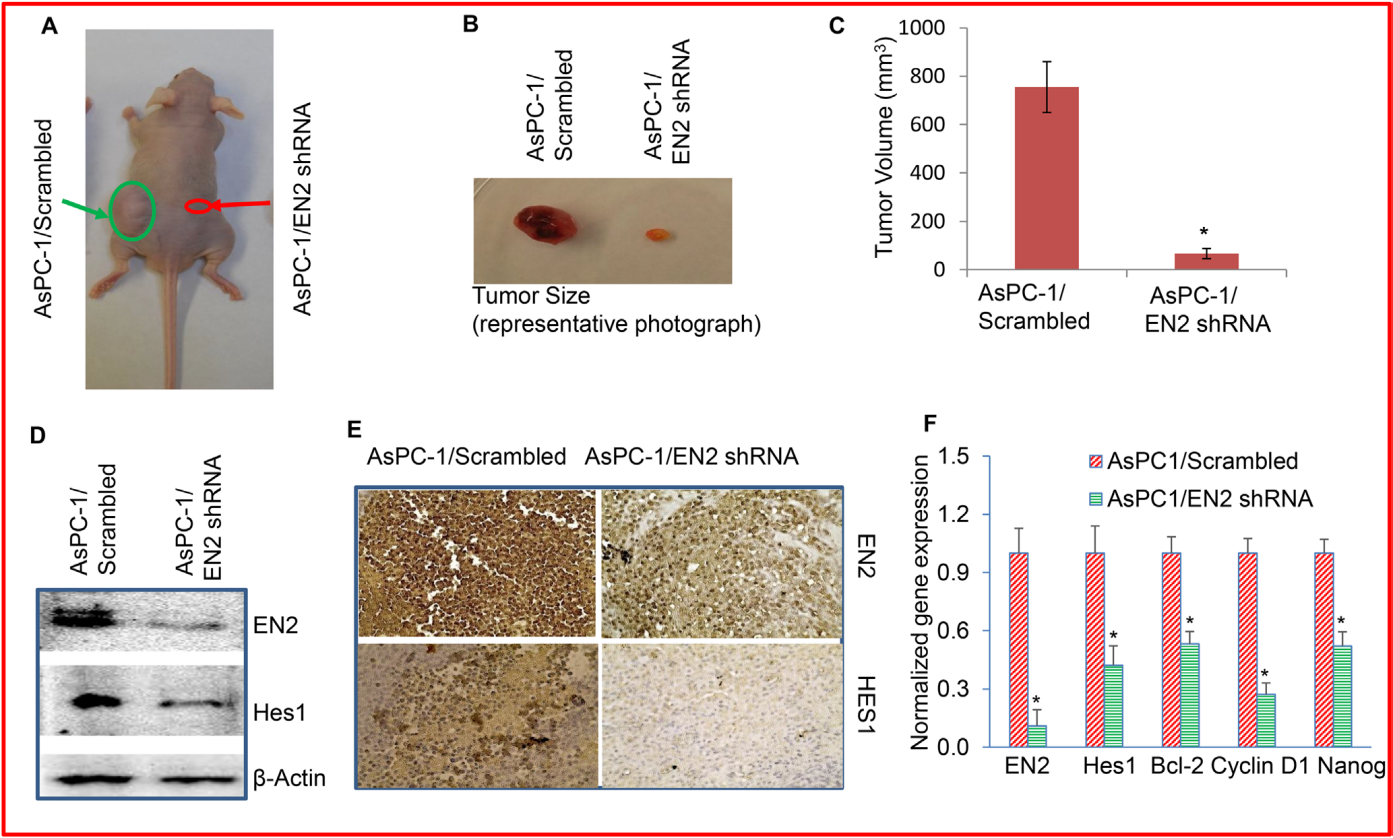
EN2 knockdown suppresses AsPC‐1 tumour growth in vivo and reduces expression of EN2 regulated genes. (A), Tumour xenograft model. AsPC‐1/Scrambled and AsPC‐1/EN2 shRNA cells (1 × 10^6^ cells mixed 1:1 with Matrigel) were injected into the dorsal left and right flanks of Balb/c nude mice, respectively. Tumour growth was monitored for 45 days. (B), Tumour size. Representative photographs of tumours derived from AsPC‐1/Scrambled and AsPC‐1/EN2 shRNA cells. (C), Tumour volume. Data represent mean (*n* = 7) ± SD. * = significantly different from scrambled control group (*p* < 0.05). (D), Expression of EN2 and Hes1 in tumour samples by WB. Tumours were extracted from AsPC‐1/Scrambled and AsPC‐1/EN2 shRNA groups, and Western blot analysis was performed to measure the expression of EN2 and Hes1. (E), Immunohistochemistry. Representative photographs for the expression of EN2 and Hes1 in tumour samples. Tumours were extracted from AsPC‐1/Scrambled and AsPC‐1/EN2 shRNA groups, and IHC was performed to measure the expression of EN2 and Hes1. (F), Expression of EN2 target genes. RNA was isolated, and the expression of EN2, Hes1, Bcl‐2, Cyclin D1 and Nanog was measured in tumour tissues by q‐RT‐PCR. GAPDH was used as an internal control. * = significantly different from scrambled control group (*p* < 0.05).

Since EN2 shRNA attenuated cancer cell proliferation, colony formation, and EMT in pancreatic cancer cells, we next examined whether EN2 depletion could also suppress tumour growth in vivo. AsPC‐1/Scrambled and AsPC‐1/EN2 shRNA cells were injected subcutaneously into BALB/c nude mice, and tumour growth was monitored over time. As shown in Figure 9A–C, tumours derived from AsPC‐1/EN2 shRNA cells were significantly smaller than those from the AsPC‐1/Scrambled control group. Consistent with these observations, EN2 expression was markedly reduced in EN2 shRNA tumours, as confirmed by Western blotting and immunohistochemistry (Figure 9D,E). Because Hes1 is a downstream Notch target, we also assessed its expression and found that Hes1 levels were significantly lower in EN2 shRNA tumours than in controls. qRT‐PCR analysis further demonstrated reduced expression of EN2, Bcl‐2, cyclin D1 and Nanog in tumour tissues from the EN2 shRNA group (Figure 9F). Together, these results indicate that EN2 promotes AsPC‐1 tumour growth in vivo, at least in part by modulating the Notch pathway and its downstream targets, as well as by regulating Nanog expression.

## 4 Discussion

Our findings position EN2 as a critical regulator of pancreatic cancer initiation, progression, and metastatic potential. By demonstrating that EN2 controls a network of genes involved in pluripotency, cell survival, cell‐cycle progression, drug resistance, and epithelial–mesenchymal transition, this study reveals a previously underappreciated breadth of EN2's influence on PDAC biology. The ability of EN2 to modulate key transcriptional programs—including c‐Myc, Oct‐4, KLF4, Nanog, Hes1, cyclin D1, Bcl‐2 and EMT‐associated cadherins—suggests that EN2 functions as a central node integrating stemness, proliferation, and invasive behaviour. These pathways are well‐recognised contributors to PDAC aggressiveness, and EN2's regulation of them provides a mechanistic explanation for its oncogenic activity.

Importantly, the convergence of these EN2‐regulated pathways aligns with hallmark features of PDAC, including its high degree of cellular plasticity, resistance to apoptosis, and propensity for early dissemination. The coordinated upregulation of stemness‐associated transcription factors, alongside pro‐survival and EMT‐related genes, suggests that EN2 may help maintain a tumour cell state that is both highly adaptable and intrinsically resistant to therapy. This multifaceted regulatory capacity positions EN2 not only as a driver of tumour progression but also as a potential determinant of therapeutic response, raising the possibility that EN2‐high tumours may represent a biologically distinct and clinically challenging PDAC subset.

Our findings demonstrate that EN2 knockdown markedly suppresses the tumorigenic properties of pancreatic cancer cells in both in vitro and in vivo settings. Silencing EN2 inhibited pancreatic cancer cell proliferation, migration, and invasion, and reduced the expression of EMT markers and key EMT‐associated transcription factors, including Snail, Slug, and Zeb1. In a pancreatic cancer xenograft mouse model, EN2 shRNA–transduced cells exhibited significantly reduced tumour growth compared with negative‐control shRNA cells. Taken together, these results provide compelling evidence that EN2 functions as an oncogenic regulator in pancreatic cancer and highlight its potential as a promising molecular target for the prevention and treatment of this highly lethal disease.

EN2 is a homeobox‐containing transcription factor belonging to the Engrailed family that has recently emerged as a novel oncogene in breast, prostate, and bladder cancers [34, 35, 36, 37, 38, 47, 48, 49]. However, its expression pattern and functional significance in pancreatic cancer have not been investigated, and its role in pancreatic tumorigenesis has remained unknown. In this study, we examined the expression and biological relevance of EN2 in pancreatic cancer and provided the first evidence supporting its oncogenic function in this malignancy. We found that EN2 is markedly upregulated in pancreatic cancer tissues and cell lines. Analysis of TCGA datasets, together with immunohistochemical validation using a pancreatic cancer tissue microarray, revealed significantly higher EN2 expression in tumour tissues than in adjacent normal tissues, and its overexpression correlated with poorer overall survival. To define the functional role of EN2, we conducted gain‐ and loss‐of‐function studies, which demonstrated that EN2 is critical for pancreatic cancer initiation, progression, and metastatic potential. These findings collectively establish EN2 as a previously unrecognised oncogenic driver in pancreatic cancer.

Overexpression of EN2 in human normal pancreatic epithelial cells induced stemness and malignant cellular transformation. EN2‐overexpressing HPNE cells exhibited increased expression of stem cell markers CD24, CD44, and CD133, as well as the pluripotency‐associated transcription factors c‐Myc and Oct4, indicating acquisition of a stem‐like phenotype. Cellular transformation was further demonstrated by enhanced proliferation, robust colony formation in soft agar, and spheroid formation in suspension—features absent in control HPNE cells transduced with an empty vector. Collectively, these findings reveal that EN2 plays a pivotal role in pancreatic cancer initiation by driving cellular transformation, promoting stemness, and sustaining pluripotency.

A notable aspect of EN2's oncogenic function is its interaction with the Notch signalling pathway, which has long been implicated in pancreatic tumorigenesis, maintenance of cancer stem‐like cells, and resistance to therapy. EN2 knockdown reduced the expression of multiple Notch‐regulated genes, including Hes1, c‐Myc and cyclin D1, suggesting that EN2 may act upstream of, or in concert with, Notch signalling to sustain proliferative and stemness‐associated transcriptional programs. This connection provides a mechanistic rationale for the observed phenotypic effects and raises the possibility that EN2 contributes to the maintenance of a stem‐like, therapy‐resistant cell population in PDAC.

In pancreatic cancer cells, EN2 upregulation was associated with epithelial–mesenchymal transition (EMT), as evidenced by increased expression of N‐cadherin and EMT‐related transcription factors, along with reduced expression of the epithelial marker E‐cadherin. To further define the role of EN2 in EMT regulation, we examined the effects of EN2 knockdown on EMT marker expression in pancreatic cancer cells. EN2 silencing resulted in a marked increase in E‐cadherin and a corresponding decrease in N‐cadherin. Similarly, EMT‐associated transcription factors were significantly suppressed in EN2 shRNA–transduced cells. These findings demonstrate that EN2 is a key regulator of EMT in pancreatic cancer and that its downregulation suppresses pancreatic cancer progression, at least in part by reversing EMT.

Functionally, EN2 depletion profoundly impaired PDAC cell proliferation, invasion, and metastatic capacity in vitro, and significantly reduced tumour growth in vivo. These phenotypic effects underscore the biological importance of EN2 and support the conclusion that EN2 is not merely a biomarker of aggressive disease but an active driver of tumour progression. This growth inhibition was associated with reduced Notch signalling activity, indicating that EN2 promotes tumour progression through a Notch‐dependent mechanism. Specifically, EN2 knockdown led to downregulation of the Notch target genes Hes1 and Cyclin D1, which are involved in cell survival and cell‐cycle progression, as well as suppression of Bcl‐2, a key regulator of drug resistance. The suppression of tumour growth in xenograft models following EN2 knockdown further highlights its potential as a therapeutic target, particularly given the limited number of actionable molecular drivers currently available in PDAC.

In conclusion, our findings identify EN2 as a novel oncogenic driver with broad regulatory influence over pathways central to PDAC biology. EN2 promotes tumour initiation, progression, and metastasis, in part through its modulation of Notch signalling and stemness‐associated gene networks, underscoring its functional importance in maintaining aggressive tumour phenotypes. These results highlight the potential clinical relevance of EN2, both as a biomarker for high‐risk disease and as a promising therapeutic target. Future studies aimed at defining the upstream regulators of EN2, mapping its direct transcriptional targets, and developing strategies to inhibit its activity will be essential for translating these insights into clinical applications. Taken together, our work demonstrates that EN2 plays a crucial role in the initiation and progression of pancreatic cancer and represents a compelling target for therapeutic intervention.

## Author Contributions

**Wei Yu:** investigation, methodology, validation, formal analysis, project administration, data curation. **Raj K. Varma:** investigation, validation, methodology, writing – original draft, formal analysis, data curation, project administration. **Varun Chandra Boinpelly:** investigation, validation, writing – review and editing, writing – original draft, data curation. **Rakesh K. Srivastava:** resources, supervision, writing – review and editing. **Emma Gombos:** investigation, methodology, validation, writing – review and editing, writing – original draft. **Yiming Ma:** investigation, methodology, validation, writing – review and editing, formal analysis, data curation. **Areej Khatri:** writing – original draft, methodology, investigation, writing – review and editing, validation. **Sharmila Shankar:** project administration, resources, supervision, writing – review and editing.

## Funding

This work was supported in part by the Department of Veterans Affairs Merit Award (BX001583 to S.S.).

## Conflicts of Interest

The authors declare no conflicts of interest.

## Data Availability

The data that support the findings of this study are available on request from the corresponding author. The data are not publicly available due to privacy or ethical restrictions.

## References

[1] W. Zheng , G. Jiang , J. G. Oparinde , et al., “Global Surge in Pancreatic Cancer Cases Driven by Ageing Populations and Modifiable Risks,” Journal of Global Health 16 (2026): 04032. 41614572 10.7189/jogh.16.04032 PMC12856964

[2] R. L. Siegel , T. B. Kratzer , N. S. Wagle , H. Sung , and A. Jemal , “Cancer Statistics, 2026,” CA: A Cancer Journal for Clinicians 76 (2026): e70043. 41528114 10.3322/caac.70043 PMC12798275

[3] B. Quoc Lam , S. K. Shrivastava , A. Shrivastava , S. Shankar , and R. K. Srivastava , “The Impact of Obesity and Diabetes Mellitus on Pancreatic Cancer: Molecular Mechanisms and Clinical Perspectives,” Journal of Cellular and Molecular Medicine 24 (2020): 7706–7716. 32458441 10.1111/jcmm.15413 PMC7348166

[4] D. Singh , G. Upadhyay , R. K. Srivastava , and S. Shankar , “Recent Advances in Pancreatic Cancer: Biology, Treatment, and Prevention,” Biochimica et Biophysica Acta 1856 (2015): 13–27. 25977074 10.1016/j.bbcan.2015.04.003

[5] S. Javed and S. E. Langley , “Importance of HOX Genes in Normal Prostate Gland Formation, Prostate Cancer Development and Its Early Detection,” BJU International 113 (2014): 535–540. 23937390 10.1111/bju.12269

[6] A. Wallen and T. Perlmann , “Transcriptional Control of Dopamine Neuron Development,” Annals of the New York Academy of Sciences 991 (2003): 48–60. 12846973 10.1111/j.1749-6632.2003.tb07462.x

[7] D. M. Wellik , “Hox Genes and Vertebrate Axial Pattern,” Current Topics in Developmental Biology 88 (2009): 257–278. 19651308 10.1016/S0070-2153(09)88009-5

[8] N. Shah and S. Sukumar , “The Hox Genes and Their Roles in Oncogenesis,” Nature Reviews. Cancer 10 (2010): 361–371. 20357775 10.1038/nrc2826

[9] S. E. McGrath , A. Michael , R. Morgan , and H. Pandha , “EN2: A Novel Prostate Cancer Biomarker,” Biomarkers in Medicine 7 (2013): 893–901. 24266821 10.2217/bmm.13.115

[10] N. Sengupta , E. Siddiqui , and F. H. Mumtaz , “Cancers of the Bladder,” Journal of the Royal Society for the Promotion of Health 124 (2004): 228–229. 15493783 10.1177/146642400412400520

[11] E. Killick , R. Morgan , F. Launchbury , et al., “Role of Engrailed‐2 (EN2) as a Prostate Cancer Detection Biomarker in Genetically High Risk Men,” Scientific Reports 3 (2013): 2059. 23792811 10.1038/srep02059 PMC3690389

[12] M. P. Marszall , W. Sroka , M. Adamowski , et al., “Engrailed‐2 Protein as a Potential Urinary Prostate Cancer Biomarker: A Comparison Study Before and After Digital Rectal Examination,” European Journal of Cancer Prevention 24 (2014): 51–60. 10.1097/CEJ.0000000000000046 25003607

[13] R. Morgan , A. Boxall , A. Bhatt , et al., “Engrailed‐2 (EN2): A Tumor Specific Urinary Biomarker for the Early Diagnosis of Prostate Cancer,” Clinical Cancer Research 17 (2011): 1090–1098. 21364037 10.1158/1078-0432.CCR-10-2410

[14] R. Morgan , R. T. Bryan , S. Javed , et al., “Expression of Engrailed‐2 (EN2) Protein in Bladder Cancer and Its Potential Utility as a Urinary Diagnostic Biomarker,” European Journal of Cancer 49 (2013): 2214–2222. 23434148 10.1016/j.ejca.2013.01.019

[15] H. Pandha , K. D. Sorensen , T. F. Orntoft , et al., “Urinary Engrailed‐2 (EN2) Levels Predict Tumour Volume in Men Undergoing Radical Prostatectomy for Prostate Cancer,” BJU International 110 (2012): E287–E292. 22583908 10.1111/j.1464-410X.2012.11208.x

[16] J. L. Ables , J. J. Breunig , A. J. Eisch , and P. Rakic , “Not(Ch) Just Development: Notch Signalling in the Adult Brain,” Nature Reviews. Neuroscience 12 (2011): 269–283. 21505516 10.1038/nrn3024 PMC3159580

[17] E. R. Andersson and U. Lendahl , “Therapeutic Modulation of Notch Signalling – Are We There Yet?,” Nature Reviews. Drug Discovery 13 (2014): 357–378. 24781550 10.1038/nrd4252

[18] R. Kopan and M. X. Ilagan , “The Canonical Notch Signaling Pathway: Unfolding the Activation Mechanism,” Cell 137 (2009): 216–233. 19379690 10.1016/j.cell.2009.03.045 PMC2827930

[19] U. Koch and F. Radtke , “Notch and Cancer: A Double‐Edged Sword,” Cellular and Molecular Life Sciences 64 (2007): 2746–2762. 17687513 10.1007/s00018-007-7164-1 PMC11136344

[20] L. Miele , T. Golde , and B. Osborne , “Notch Signaling in Cancer,” Current Molecular Medicine 6 (2006): 905–918. 17168741 10.2174/156652406779010830

[21] L. Kopper and M. Hajdu , “Tumor Stem Cells – A Possible Scenario,” Magyar Onkologia 50 (2006): 101–106. 16888672

[22] G. Weinmaster and J. A. Fischer , “Notch Ligand Ubiquitylation: What Is It Good for?,” Developmental Cell 21 (2011): 134–144. 21763614 10.1016/j.devcel.2011.06.006 PMC3156059

[23] Q. Shi , C. Xue , Y. Zeng , et al., “Notch Signaling Pathway in Cancer: From Mechanistic Insights to Targeted Therapies,” Signal Transduction and Targeted Therapy 9 (2024): 128. 38797752 10.1038/s41392-024-01828-x PMC11128457

[24] P. Ranganathan , K. L. Weaver , and A. J. Capobianco , “Notch Signalling in Solid Tumours: A Little Bit of Everything but Not All the Time,” Nature Reviews. Cancer 11 (2011): 338–351. 21508972 10.1038/nrc3035

[25] S. Zhou and S. D. Hayward , “Nuclear Localization of CBF1 Is Regulated by Interactions With the SMRT Corepressor Complex,” Molecular and Cellular Biology 21 (2001): 6222–6232. 11509665 10.1128/MCB.21.18.6222-6232.2001 PMC87339

[26] B. Zhou , W. Lin , Y. Long , et al., “Notch Signaling Pathway: Architecture, Disease, and Therapeutics,” Signal Transduction and Targeted Therapy 7 (2022): 95. 35332121 10.1038/s41392-022-00934-y PMC8948217

[27] W. Yu , Y. Ma , S. Shankar , and R. K. Srivastava , “Role of SATB2 in Human Pancreatic Cancer: Implications in Transformation and a Promising Biomarker,” Oncotarget 7 (2016): 57783–57797. 27472393 10.18632/oncotarget.10860 PMC5295389

[28] W. Yu , Y. Ma , S. Shankar , and R. K. Srivastava , “Chronic Ethanol Exposure of Human Pancreatic Normal Ductal Epithelial Cells Induces Cancer Stem Cell Phenotype Through SATB2,” Journal of Cellular and Molecular Medicine 22 (2018): 3920–3928. 29761897 10.1111/jcmm.13666 PMC6050497

[29] W. Yu , Y. Ma , S. Shankar , and R. K. Srivastava , “SATB2/Beta‐Catenin/TCF‐LEF Pathway Induces Cellular Transformation by Generating Cancer Stem Cells in Colorectal Cancer,” Scientific Reports 7 (2017): 10939. 28887549 10.1038/s41598-017-05458-y PMC5591219

[30] W. Yu , R. Srivastava , S. Srivastava , Y. Ma , S. Shankar , and R. K. Srivastava , “Oncogenic Role of SATB2 In Vitro: Regulator of Pluripotency, Self‐Renewal, and Epithelial‐Mesenchymal Transition in Prostate Cancer,” Cells 13 (2024): 13. 10.3390/cells13110962 PMC11171950 38891096

[31] R. Nanta , D. Kumar , D. Meeker , et al., “NVP‐LDE‐225 (Erismodegib) Inhibits Epithelial‐Mesenchymal Transition and Human Prostate Cancer Stem Cell Growth in NOD/SCID IL2Rgamma Null Mice by Regulating Bmi‐1 and microRNA‐128,” Oncogene 2 (2013): e42. 10.1038/oncsis.2013.5 PMC3641359 23567619

[32] W. Yu , Y. Ma , A. C. Ochoa , S. Shankar , and R. K. Srivastava , “Cellular Transformation of Human Mammary Epithelial Cells by SATB2,” Stem Cell Research 19 (2017): 139–147. 28167342 10.1016/j.scr.2017.01.011

[33] S. Shankar , T. R. Singh , X. Chen , H. Thakkar , J. Firnin , and R. K. Srivastava , “The Sequential Treatment With Ionizing Radiation Followed by TRAIL/Apo‐2 L Reduces Tumor Growth and Induces Apoptosis of Breast Tumor Xenografts in Nude Mice,” International Journal of Oncology 24 (2004): 1133–1140. 15067334

[34] S. E. McGrath , N. Annels , T. K. Madhuri , et al., “Engrailed‐2 (EN2) ‐ a Novel Biomarker in Epithelial Ovarian Cancer,” BMC Cancer 18 (2018): 943. 30285763 10.1186/s12885-018-4816-5 PMC6171236

[35] S. E. McGrath , A. Michael , R. Morgan , and H. Pandha , “EN2 in Prostate Cancer,” Advances in Clinical Chemistry 71 (2015): 47–76. 26411411 10.1016/bs.acc.2015.06.002

[36] M. I. D. Rosa , E. R. Dondossola , M. C. M. Alexandre , K. Madeira , F. A. Cardoso , and A. J. Grande , “Urinary EN‐2 to Predict Prostate Cancer: Systematic Review and Meta‐Analysis,” Revista da Associacao Medica Brasileira (1992) 63 (2017): 656–661. 28977093 10.1590/1806-9282.63.07.656

[37] D. Chen and R. Yin , “Engrailed 2 Facilitates Progression of Triple‐Negative and HER2‐Enriched Breast Cancer by Binding to Enhancer Region of Tenascin‐C,” Discover Oncology 15 (2024): 705. 39581954 10.1007/s12672-024-01471-6 PMC11586318

[38] S. K. Bose , R. S. Bullard , and C. D. Donald , “Oncogenic Role of Engrailed‐2 (en‐2) in Prostate Cancer Cell Growth and Survival,” Translational Oncogenomics 3 (2008): 37–43. 21566742 PMC3022358

[39] G. Nguyen‐Ba and P. Vasseur , “Epigenetic Events During the Process of Cell Transformation Induced by Carcinogens (Review),” Oncology Reports 6 (1999): 925–932. 10373683 10.3892/or.6.4.925

[40] M. L. Franco‐Chuaire , S. C. Magda Carolina , and L. Chuaire‐Noack , “Epithelial‐Mesenchymal Transition (EMT): Principles and Clinical Impact in Cancer Therapy,” Investigación Clínica 54 (2013): 186–205. 23947008

[41] J. P. Thiery , H. Acloque , R. Y. Huang , and M. A. Nieto , “Epithelial‐Mesenchymal Transitions in Development and Disease,” Cell 139 (2009): 871–890. 19945376 10.1016/j.cell.2009.11.007

[42] W. Yu , Y. Ma , S. K. Shrivastava , R. K. Srivastava , and S. Shankar , “Chronic Alcohol Exposure Induces Hepatocyte Damage by Inducing Oxidative Stress, SATB2 and Stem Cell‐Like Characteristics, and Activating Lipogenesis,” Journal of Cellular and Molecular Medicine 26 (2022): 2119–2131. 35152538 10.1111/jcmm.17235 PMC8980954

[43] C. Brown , S. Srivastava , R. Srivastava , et al., “SATB2 Induces Malignant Transformation and Cancer Stem Cell Characteristics, and Inhibition of Its Expression Reverses Drug Resistance in Mesothelioma,” Cells 15 (2026): 283–299. 41677646 10.3390/cells15030283 PMC12896865

[44] R. Derynck and R. A. Weinberg , “EMT and Cancer: More Than Meets the Eye,” Developmental Cell 49 (2019): 313–316. 31063750 10.1016/j.devcel.2019.04.026 PMC7672963

[45] C. L. Chaffer , B. P. San Juan , E. Lim , and R. A. Weinberg , “EMT, Cell Plasticity and Metastasis,” Cancer Metastasis Reviews 35 (2016): 645–654. 27878502 10.1007/s10555-016-9648-7

[46] M. Huang , S. N. Tang , G. Upadhyay , et al., “Rottlerin Suppresses Growth of Human Pancreatic Tumors in Nude Mice, and Pancreatic Cancer Cells Isolated From Kras(G12D) Mice,” Cancer Letters 353 (2014): 32–40. 25050737 10.1016/j.canlet.2014.06.021

[47] C. Y. Lai , Y. Xu , G. S. Yu , et al., “Engrailed‐2 Might Play an Anti‐Oncogenic Role in Clear‐Cell Renal Cell Carcinoma,” Journal of Molecular Histology 47 (2016): 229–237. 26948025 10.1007/s10735-016-9665-4

[48] A. D. Ranzi , J. N. da Silva , T. M. Graziottin , N. Annels , and C. G. Bica , “Immunohistochemistry Biomarkers in Nonmuscle Invasive Bladder Cancer,” Applied Immunohistochemistry & Molecular Morphology (2015). 178‐183. 10.1097/PAI.0000000000000280 26574637

[49] N. L. Martin , M. K. Saba‐El‐Leil , S. Sadekova , S. Meloche , and G. Sauvageau , “EN2 Is a Candidate Oncogene in Human Breast Cancer,” Oncogene 24 (2005): 6890–6901. 16007149 10.1038/sj.onc.1208840

